# Correlated quantum machines beyond the standard second law

**DOI:** 10.1126/sciadv.adw8462

**Published:** 2025-10-10

**Authors:** Milton Aguilar, Eric Lutz

**Affiliations:** Institute for Theoretical Physics I, University of Stuttgart, D-70550 Stuttgart, Germany.

## Abstract

The laws of thermodynamics strongly restrict the performance of thermal machines. Standard thermodynamics, initially developed for uncorrelated macroscopic systems, does not hold for microscopic systems correlated with their environments. We here derive an exact formula for the efficiency of any cyclically driven quantum engine by using generalized laws of quantum thermodynamics that account for all possible correlations between all involved parties, including initial correlations. Furthermore, we demonstrate the existence of two basic modes of engine operation: the usual thermal case, where heat is converted into work, and an athermal regime, where work is extracted from entropic resources, such as system-bath correlations. In the latter regime, the efficiency is not bounded by the usual Carnot formula. Our results provide a unified formalism to determine the efficiency of correlated microscopic quantum machines.

## Teaser

Quantum engines can attain efficiencies higher than Carnot’s by using entropic resources.

## INTRODUCTION

Thermodynamics offers a powerful framework to describe the equilibrium properties of macroscopic systems. By providing quantitative relationships between observable quantities, it allows one to predict the state of arbitrary systems when external parameters are varied (*1*). An important application of the formalism is the study of the interconversion of different energy forms, such as mechanical, chemical, and thermal energies. In particular, the laws of thermodynamics restrict the efficiency of heat-work conversion in cyclic processes (*1*). The maximum achievable value of the efficiency, defined as the ratio of work output and heat input, of any heat engine coupled to two thermal baths is thus given by the Carnot formula, $\eta_{C}=1-T_{c}/T_{h}$, where $T_{c,h}$ are the respective temperatures of the cold and hot heat reservoirs (*1*).

Despite its successes, standard thermodynamics relies on the fundamental hypothesis that system and baths are uncorrelated (*2*). This assumption is well justified for large classical systems that weakly interact with their reservoirs, since system-bath coupling energies, which typically scale like the surface of the systems, are usually much smaller than their internal energies, which scale like their volume (*2*). However, this condition is often violated for microscopic quantum systems owing to the presence of strong interactions (*3*) and/or quantum correlations (*4*) between system and reservoirs. In particular, system and baths can be entangled, even for weak coupling, below a critical temperature (*5*–*10*). As a consequence, the usual laws of macroscopic thermodynamics no longer apply in this regime and need to be generalized (*11*–*21*). While several methods to include correlations between a system and one or two reservoirs have been put forward (*11*–*21*), including an information-theoretic framework (*13*), a repeated interaction scheme (*15*), or a “Hamiltonian of mean force” approach (*19*, *20*), a general formalism that allows one to explicitly account for all possible quantum correlations, within the system, between the system and many baths, as well as between the baths is missing.

Quantum machines that convert one form of energy into another, such as engines and refrigerators (*22*, *23*), aim at harnessing quantum effects to improve their performance (*24*–*27*). Quantum phenomena, including quantum coherence, quantum correlations and squeezing, may indeed strongly impact thermodynamic processes, and have the potential to be exploited as a resource (*24*–*27*). Initial correlations have, for example, been found to induce heat flow reversals (*16*, *17*), highlighting the need to generalize the second law in this situation (*28*, *29*). In addition, coherence (*30*, *31*), correlations (*32*, *33*), and squeezing (*34*, *35*), have been shown to increase the efficiency of cyclic machines, and even surpass the Carnot bound in some instances. However, a unified framework allowing one to describe the performance of quantum correlated machines is currently not available. Especially, their maximum possible efficiency in such broad setting is currently unknown.

We here formulate universally applicable extensions of the first and second laws for a generic quantum system subjected to periodic driving and coupled to an arbitrary number of reservoirs via general, not necessarily energy conserving, interactions. We obtain an exact, generalized Clausius equality that accounts for all possible quantum correlations at all times. We uncover a nonequilibrium contribution to the entropy balance that, unlike the entropy production, can be negative. We show that a negative sign is associated with an operation regime in which engines extract work out of entropic resources, such as system-bath correlations, instead of heat. We moreover derive a generalized formula for their maximum efficiency that contains this distinct entropic contribution, and demonstrate that it may exceed the standard Carnot limit. Last, we illustrate our results by analyzing the performance of a two-oscillator quantum engine (*36*–*38*).

## RESULTS

### Generalized laws of quantum thermodynamics

We consider a generic quantum system S that interacts with an arbitrary number of reservoirs $R_{j}$, with Hamiltonians $H_{j}$. We assume that the initial state $\rho_{j}\left(0\right)$ of each bath can be assigned a temperature $T_{j}$, such that its mean energy is that of a thermal state $\rho_{j}^{\text{th}}$ at the same temperature, $\text{tr}\left[\rho_{j}\left(0\right)H_{j}\right]=\text{tr}\left[\rho_{j}^{\text{th}}H_{j}\right]$; this is the most common approach to assign a temperature to reservoirs that are not necessarily in equilibrium (*39*). To account for correlations within the system, we divide it into a collection of noninteracting subsystems $S_{i}$ with Hamiltonians $H_{i}$; each subsystem may include many interacting parts. In the sequel, we will use the indices *i* (*j*) to refer to quantities related to the subsystems (baths). The total Hamiltonian is then $H_{\text{tot}}\left(t\right)=\sum_{i}H_{i}\left(t\right)+\sum_{j}H_{j}+\sum_{i,j}H_{\mathrm{ij}}\left(t\right)$, where $H_{\mathrm{ij}}\left(t\right)$ is the time-dependent interaction between subsystem *i* and bath *j*. We stress that we do not take it to be energy preserving (*40*), implying that we are not limited to the weak-coupling regime. We additionally focus on cyclic processes with periodic system Hamiltonians, $H_{i}\left(t\right)=H_{i}\left(t+\tau \right)$, with period τ.

We begin by writing down a generalized first law of thermodynamics. To that end, we employ standard definitions of work and heat valid for generic system-bath couplings (*41*, *42*). The total work extracted during a cycle is evaluated as $W=\int_{t}^{t+\tau }{\mathrm{dt}}^{\prime }\langle \partial_{t^{\prime }}H_{\text{tot}}\left(t^{\prime }\right)\rangle$ (*21*). Since the dynamics of the total system is unitary, the energy change is solely due to the time dependence in the Hamiltonian, which may thus be identified as work (*43*). This approach naturally avoids the problem of the partitioning of the system-bath interaction (*44*–*47*). Using the Ehrenfest theorem for $H_{\text{tot}}\left(t\right)$ (*48*), one obtains the exact energy balance (Supplementary Text)

$$W=\sum_{j}Q_{j}+\Delta U$$ (1)

where $Q_{j}\;=\;\langle H_{j}\rangle \left(t+\tau \right)\;-\;\langle H_{j}\rangle \left(t\right)$ is the heat absorbed by reservoir $R_{j}$, and $\begin{array}{c} \Delta U=\sum_{i}\Delta U_{i}=\sum_{i}\left[\langle H_{i}+\sum_{j}H_{\mathrm{ij}}\rangle \left(t+\tau \right)-\langle H_{i}+\;\sum_{j}H_{\mathrm{ij}}\rangle \left(t\right)\right] \end{array}$ is the change of the energy of the compound system, including the interaction energy. Expression 1 thus contains all the different forms of energy exchange, including the contribution originating from the modulation of the system-bath coupling. The standard first law for cycles (*1*), $W_{S}=\sum_{j}Q_{j}$, which relates the work produced by the system, $W_{S}=\sum_{i}\int_{t}^{t+\tau }{\mathrm{dt}}^{\prime }\langle \partial_{t^{\prime }}H_{i}\left(t^{\prime }\right)\rangle$, to the total heat is recovered when the internal energy remains constant over one period, and the system-bath coupling is energy conserving on average, $\sum_{i,j}\int_{t}^{t+\tau }{\mathrm{dt}}^{\prime }\langle [H_{\mathrm{ij}}\left(t^{\prime }\right),H_{i}\left(t^{\prime }\right)+H_{j}]\rangle =0$ (*40*). Note that a periodic Hamiltonian, $H_{i}\left(t\right)=H_{i}\left(t+\tau \right)$, does not necessarily imply a periodic averaged Hamiltonian, $\langle H_{i}\rangle \left(t\right)\neq \langle H_{i}\rangle \left(t+\tau \right)$, meaning that in general ΔU≠0 over one cycle. This is, for example, the case for systems that are coupled to finite reservoirs for which the state $\rho_{i}\left(t\right)$ of subsystem *i* is not periodic (*49*–*54*).

A generalized second law can be similarly obtained by computing the change of the von Neumann entropy, S(ρ)=−tr[ρln(ρ)], for each subsystem and each bath over one cycle. Using the unitarity of the total time evolution, we have $\sum_{i}\Delta S\left(\rho_{i}\right)+\sum_{j}\Delta S\left(\rho_{j}\right)=\Delta I\left(S,R\right)+\Delta C\left(S\right)+\Delta C\left(R\right)$, where $I\left(S,R\right)\;=\;S\left(\rho_{S}\right)\;+\;S\left(\rho_{R}\right)\;-\;S\left(\rho_{SR}\right)$ is the mutual information between the system S and the collection R of all reservoirs (*55*). The total correlations, $C\left(S\right)=\;\sum_{i}S\left(\rho_{i}\right)\;-S\left(\rho_{S}\right)$ and $C\left(R\right)=\sum_{j}S\left(\rho_{j}\right)-S\left(\rho_{R}\right)$, are moreover multivariate extensions of the mutual information (*56*, *57*) that quantify correlations among all the subsystems and among all the baths. The above equality can be understood as a conservation law relating the entropies of each party [$S\left(\rho_{i}\right)$ and $S\left(\rho_{j}\right)$] and the correlations [I(S,R), C(S), and C(R)] between them. It can be rewritten in the form (Supplementary Text)

$$\sum_{i}\Delta S\left(\rho_{i}\right)+\sum_{j}\frac{Q_{j}}{{\mathrm{kT}}_{j}}=\Delta \Sigma$$ (2)

where ΔΣ=Σ(t+τ)−Σ(t) with $\Sigma =I\left(S,R\right)+C\left(S\right)+C\left(R\right)+\sum_{j}D\left(\rho_{j}\|\rho_{j}^{\text{th}}\right)$ (*k* denotes the Boltzmann constant). The last sum contains the relative entropy, $D\left(\rho_{j}\|\rho_{j}^{\text{th}}\right)=\text{Tr}\rho_{j}\text{ln}\rho_{j}-\text{Tr}\rho_{j}\text{ln}\rho_{j}^{\text{th}}$ (*55*), between the reduced state $\rho_{j}$ of reservoir $R_{j}$ and the associated reference thermal state, $\rho_{j}^{\text{th}}=\text{exp}\left(-H_{j}/{\mathrm{kT}}_{j}\right)/Z_{j}$ with partition function $Z_{j}$. The term Σ(t+τ) is the nonequilibrium entropy produced during the cycle of duration τ (*4*), here expressed as the amount of correlations created between system and baths, as well as within the system and among the baths. It is nonnegative and hence quantifies the irreversibility of the process (*4*).

The general form (Eq. 2) of the entropy balance takes into account all possible sources of irreversibility, including the noncyclic behavior of the system, the displacement out of local thermal equilibrium of the reservoirs, and the creation (or destruction) of classical and quantum correlations between all parties. It thus extends previous microscopic generalizations of the second law that were obtained in the absence of initial correlations (*41*, *42*). It contains, in particular, an entropic contribution Σ(t) related to the presence of correlations at the beginning of a cycle. Whereas Σ≥0, the difference ΔΣ has no definite sign: ΔΣ>0 signals irreversible losses associated with the overall creation of correlations (*41*, *42*), while ΔΣ<0 reveals that work may be gained from available correlations by reducing them (*58*–*62*). The condition ΔΣ<0 can therefore be regarded as an indicator of an entropic resource. Equation 2 provides a unified formalism enabling the investigation of both processes on the same footing. Standard macroscopic thermodynamics completely neglects initial correlations. In that case, Eq. 2 implies the usual second law, $\Delta S+\sum_{j}Q_{j}/{\mathrm{kT}}_{j}\geq 0$ (*1*). By contrast, in the presence of initial correlations, Eq. 2 can lead to an inverted entropy balance, $\Delta S+\sum_{j}Q_{j}/{\mathrm{kT}}_{j}\leq 0$. We note that an inverted entropy balance also occurs for initially isolated equilibrium states that dissipatively evolve into a nonthermal state (*63*–*66*).

### Generalized efficiency

The efficiency η, defined as the ratio of the work produced by an engine and the energy needed to run it over one cycle (*1*), can be evaluated by combining the first and second laws, Eqs. 1 and 2 (Supplementary Text). We obtain the total work for any correlated quantum engine

$$W=\sum_{j}\eta_{j}Q_{j}+{\mathrm{kT}}_{\text{min}}\Delta \sigma$$ (3)

where we have introduced ${\mathrm{kT}}_{\text{min}}\Delta \sigma ={\mathrm{kT}}_{\text{min}}\Delta \Sigma +\sum_{i}\Delta F_{i}$, with $F_{i}=U_{i}-{\mathrm{kT}}_{\text{min}}S\left(\rho_{i}\right)$ the generalized free energy (*41*, *42*). The quantity σ(t+τ) represents the total entropy production during the cycle that now also accounts for thermalization processes occurring within the parts of system when the variation $\Delta D\left(\rho_{i}\|\rho_{i}^{\text{th}}\right)\neq 0$. It reduces to Σ(t+τ) for an ideal cyclic behavior of subsystem $\rho_{i}$ which corresponds to $\Delta F_{i}=0$. On the other hand, σ(t) is related to all the correlations (and displacements from equilibrium) present at the beginning of a cycle. The local Carnot efficiency, $\eta_{j}=1-T_{\text{min}}/\;T_{j}$, is further related to the heat flow between the reservoir at the smallest temperature, $T_{\text{min}}={\text{min}}_{j}\left\{T_{j}\right\}$, and the one at temperature $T_{j}$. Contrary to conventional macroscopic heat engines, whose sole energy source is the heat $Q_{j}$ absorbed from bath *j*, a microscopic correlated engine might also extract work from a change of the energy *U* of the system, either from a variation of the energy of subsystem *i* or of the bath coupling energies. In small systems, these energy changes can be positive or negative along individual realizations, owing to thermal and/or quantum fluctuations. We therefore define the corresponding contributions that originate from these two energy sources as $Q^{\text{in}}=\sum_{j}\left(|\;Q_{j}\;|-Q_{j}\right)\;/\;2$ and $\Delta U^{\text{in}}=\sum_{i}\left(|\;\Delta U_{i}\;|-\Delta U_{i}\right)\;/\;2$, where the label “in” refers to the energy that flows into the engine. The generalized efficiency $\eta =-W\;/\;\left(Q^{\text{in}}+\Delta U^{\text{in}}\right)$ then reads

$$\eta =\gamma \left(\eta_{\text{th}}-\frac{{\mathrm{kT}}_{\text{min}}\Delta \sigma }{Q^{\text{in}}}\right)$$ (4)

where $\gamma ={\left[1+\Delta U^{\text{in}}/Q^{\text{in}}\right]}^{-1}\leq 1$ is a parameter that assesses the fraction of the energy used to run the engine that comes from either the working substance or the interaction with the baths, versus the fraction that stems from the reservoirs in the form of heat. The efficiency $\eta_{\text{th}}=-\sum_{j}\eta_{j}Q_{j}/\;Q^{\text{in}}$ is the thermal efficiency of a reversible engine whose working substance is in a cyclic state with constant system-bath interactions. If all temperatures $T_{j}$ are positive, $\eta_{\text{th}}$ is upper bounded by the Carnot efficiency, $\eta_{\text{th}}\leq \eta_{C}=1-T_{\text{min}}/T_{\text{max}}$, where $T_{\text{max}}$ is the largest bath temperature. We note that it is equal to $\eta_{C}$ if and only if all the reservoirs from which heat flows out ($Q_{j}<0$) are at temperature $T_{\text{max}}$, and all reservoirs from which heat flows in ($Q_{j^{\prime }}>0$) are at temperature $T_{\text{min}}$. This is clearly the case for a reversible engine operating between two thermal reservoirs (*1*).

According to Eq. 4, the efficiency of any engine operating between many reservoirs is upper bounded by

$$\eta \leq \eta_{C}-\frac{{\mathrm{kT}}_{\text{min}}\Delta \sigma }{Q^{\text{in}}}$$ (5)

The Carnot efficiency $\eta_{C}$ may hence be surpassed if entropic resources are present at the beginning of a cycle such that Δσ<0. This result should not be regarded as a violation of the standard second law, but as its extension to situations where the latter does no longer apply. In standard thermodynamics, the maximum efficiency of a heat engine operating by two thermal baths is indeed given by $\eta_{C}-{\mathrm{kT}}_{\text{min}}\Sigma /Q^{\text{in}}$ (*67*), which is always smaller than $\eta_{C}$ since Σ≥0. This result follows from the general expression 5 in the absence of initial correlations (and displacements from equilibrium). A special case of Eq. 5 has recently been simulated experimentally using a spin-1/2 SWAP engine (*62*).

### Characterizing entropic resources

Equation 4 is exact and accounts for all possible correlations in the system-plus-baths ensemble, including displacements from equilibrium in both system and reservoirs. It hence allows one to evaluate/engineer entropic resources and compute/optimize the efficiency of any quantum engine from the knowledge of the microscopic Hamiltonians. This should be useful in the study/design of high-performance quantum engine models. Like in standard thermodynamics, where, for instance, the efficiency of an ideal Otto cycle explicitly depends on the heat capacity of the working medium (*1*), the numerical values of the parameters appearing in Eq. 4, such as η, $\eta_{\text{th}}$, and γ, will depend on the specific details of the considered device. However, from a macroscopic point of view, the efficiency η follows from the knowledge of the macroscopic energy fluxes in and out of the engine, which can, in principle, be determined experimentally (*62*). Likewise, while the nonequilibrium entropy production is known to be microscopically given by the amount of correlations built between (a single) system and baths during one cycle (*41*, *42*), it is macroscopically equal to $\sum_{j}Q_{j}/{\mathrm{kT}}_{j}=-\Sigma$ for a perfect cycle with $\sum_{i}\Delta S\left(\rho_{i}\right)=0$ (*1*). In the absence of initial correlations, the entropy production can therefore be evaluated from macroscopic quantities, like temperature and heat, without having to directly determine correlation measures, such as the system-bath mutual information, which can be experimentally challenging. Similarly, still for a perfect cycle, the difference ΔΣ may be inferred from $\sum_{j}Q_{j}/{\mathrm{kT}}_{j}=\Delta \Sigma$, in the presence of initial correlations. In the more general case of nonperfect cycles, $\sum_{i}\Delta S\left(\rho_{i}\right)\neq 0$, the entropic resource Δσ can be obtained from *W*, $Q_{j}$, and $T_{j}$ via Eq. 3, again without having to assess any microscopic correlation term. In that regard, it is interesting to further note that experimental methods to detect system-reservoir correlations from measurements of the system alone are currently being developed (*68*–*71*).

### Athermal engine operation

Additional insight into the physical role of entropic resources can be gained by rewriting Eq. 4 as

$$\frac{{\mathrm{kT}}_{\text{min}}\Delta \sigma }{\sum_{j}\eta_{j}Q_{j}}=\frac{\eta }{{\gamma \eta }_{\text{th}}}-1$$ (6)

Equation 6 indicates that two regimes of engine operation should be distinguished: (i) If $\eta /\;{\gamma \eta }_{\text{th}}<2$, the engine produces work by mostly converting heat $Q_{j}$ from the reservoirs, that is, by exploiting thermal resources, like common macroscopic engines. (ii) On the other hand, if $\eta /\;{\gamma \eta }_{\text{th}}>2$, the engine dominantly extracts work from athermal resources, such as correlations, displacements from equilibrium, or interactions with the baths, quantified by Δσ. This particular regime is possible for correlated microscopic engines.

**Fig. 1.**
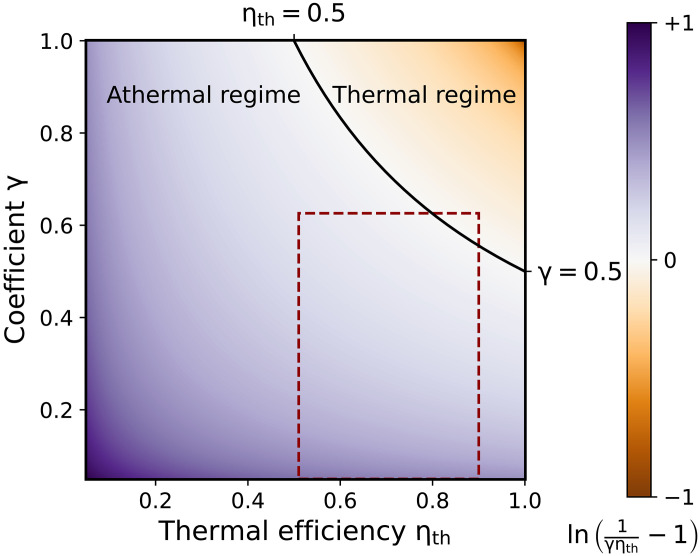
Operation regimes of correlated engines. Depending on the value of the ratio, Eq. 6, of thermal and athermal contributions, correlated engines may produce work by converting heat (thermal regime) or entropic resources, such as correlations (athermal regime). The plot shows the logarithm of the upper bound of Eq. 6 (normalized to [−1,1]), as a function of the thermal efficiency $\eta_{\text{th}}$ and the coefficient γ. Engines are guaranteed to run in the thermal regime when $1\;/\;{\gamma \eta }_{\text{th}}>2$ (solid line). The dashed rectangle indicates the region explored by the two-oscillator engine example of Fig. 3.

To make model-independent statements, we next analyze the upper bound, $1\;/\;{\gamma \eta }_{\text{th}}-1$, of Eq. 6, by using the fact that the efficiency η, which depends on the details of the engine, is always smaller than one. Figure 1 presents the normalized logarithm of $1\;/\;{\gamma \eta }_{\text{th}}-1$ as a function of the parameter γ and of the thermal efficiency $\eta_{\text{th}}$. The black solid line indicates the limiting value $1\;/\;{\gamma \eta }_{\text{th}}=2$. An engine is only guaranteed to run in the thermal regime provided that γ>1/2 and $\eta_{\text{th}}>1\;/\;2$. The first condition is expected from the definition of the parameter γ; it simply says that the engine should be predominantly fueled by heat. However, unexpectedly, this criterion is not enough. The nontrivial condition $\eta_{\text{th}}>1\;/\;2$ reveals that, since $\eta_{\text{th}}\leq \eta_{C}$, the temperature of the hottest reservoir should be at least twice the temperature of the colder one. Otherwise, work production could still be dominated by athermal resources, even if γ>1/2.

Physical intuition about the athermal regime may be developed by considering the simple example of a (single system) engine operating between two reservoirs in the weak-coupling limit. In the high-temperature (classical) domain, thermalization of the system with a bath (either hot or cold) at the beginning of a cycle does not create any (substantial) system-bath correlations. In this thermal regime, the engine is solely fueled by heat. By contrast, in the low-temperature (quantum) domain, thermalization will in general lead to initial system-bath correlations (*5*–*10*) that may be quantified by a nonzero mutual information $I\left(S,R_{j}\right)$. An engine will run in the athermal regime, when primarily fueled by these initial correlations, instead of heat. However, when $\eta_{\text{th}}>1\;/\;2$, that is, $T_{h}\geq 2T_{c}$, the heat flowing between the two baths (which is proportional to the temperature difference) can become large enough that the engine is again predominantly fueled by heat, instead of correlations. The presence of initial correlations does hence not necessarily imply that they will be dominantly exploited. The condition $\eta_{\text{th}}>1\;/\;2$ is necessary, but not sufficient, to be in the thermal regime, as we discuss below. We additionally mention that another way to create initial system-bath correlations is to move to the strong-coupling limit.

### Two-oscillator quantum engine

**Fig. 2.**
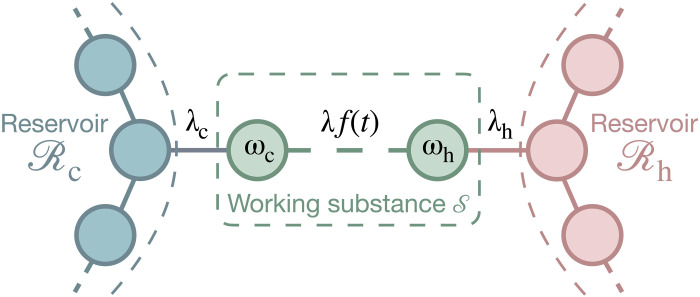
Two-oscillator quantum engine. The working substance S is composed of two harmonic oscillators, with respective frequencies $\omega_{c}$ and $\omega_{h}$, each coupled to its own reservoir, $R_{c}$ and $R_{h}$, with temperatures $T_{c}$ and $T_{h}$, and coupling constants $\lambda_{c}$ and $\lambda_{h}$. Work is produced by periodically switching the oscillator interaction λf(t) on and off. The oscillators thermalize with their respective baths during the off phases, leading to the creation of correlations between them.

As an illustration, we now consider a quantum engine whose working substance consists of two harmonic oscillators (with frequencies $\omega_{c}$ and $\omega_{h}$), each coupled to its own reservoir $R_{c}$ and $R_{h}$ (with respective temperatures $T_{c}$ and $T_{h}$ and respective coupling constants $\lambda_{c}$ and $\lambda_{h}$) (Fig. 2) (*36*–*38*). The engine is operated by periodically switching the interaction λf(t) between the oscillators on and off, extracting work. The potential *f*(*t*) is taken to be a bump function with unit amplitude (Materials and Methods). The interaction is turned on for a short time {$t_{\text{on}}=41\left[2\pi /\left(\omega_{h}-\omega_{c}\right)+1\right]/40$} and turned off for a longer time {$t_{\text{off}}=5\left[2\pi /\left(\omega_{h}-\omega_{c}\right)+1\right]$} to allow the oscillators to thermalize with their baths. We numerically analyze the performance of the engine by modeling the finite reservoirs by an ensemble of 300 harmonic oscillators each (*52*). The initial state is taken to be the direct product, $\rho \left(0\right)=\rho_{c}⊗\rho_{h}$, of the joint thermal states $\rho_{c,h}$ of each oscillator with its respective reservoir. The thermalization between each oscillator and its associated bath leads to the built-up of correlations between the two (*5*–*10*), implying the creation of athermal resources with σ(t)≠0 at the beginning of a cycle.

**Fig. 3.**
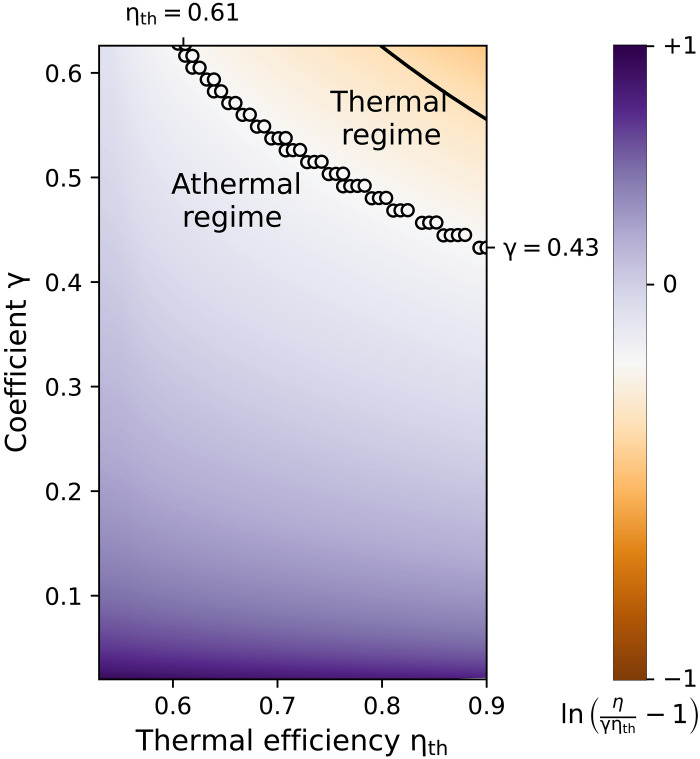
Operation regimes of the two-oscillator engine. The plot shows the (normalized) logarithm of Eq. 6, after the first cycle. The black circles indicate the exact boundary between thermal and athermal regimes given by $\eta \;/\;{\gamma \eta }_{\text{th}}=2$, whereas the solid line corresponds to the device-independent upper bound of Fig. 1. Parameters are $\omega_{c}=1$, $\omega_{h}=2$, λ=0.08, $\lambda \;/\;15\leq \lambda_{c}=\lambda_{h}\leq \lambda \;/\;2$, $T_{c}=0.8$, and $1.7\leq T_{h}\leq 8$.

Whether the engine can exploit these athermal resources depends on the chosen parameter values. Figure 3 shows the normalized logarithm of $\eta /{\gamma \eta }_{\text{th}}-1$ when the temperature $T_{h}$ of the hot bath and the coupling constants $\lambda_{c}$ and $\lambda_{h}$ are varied in the domain where the device runs as an engine (all other parameters are kept fixed); this, in turn, modifies the values of $\eta_{\text{th}}$ and γ. The athermal regime (violet) clearly dominates the thermal regime (orange), and occupies more than 87% of the parameter space; the black circles represent the numerically determined boundary between the two regimes, while the black line is the upper bound shown in Fig. 1.

**Fig. 4.**
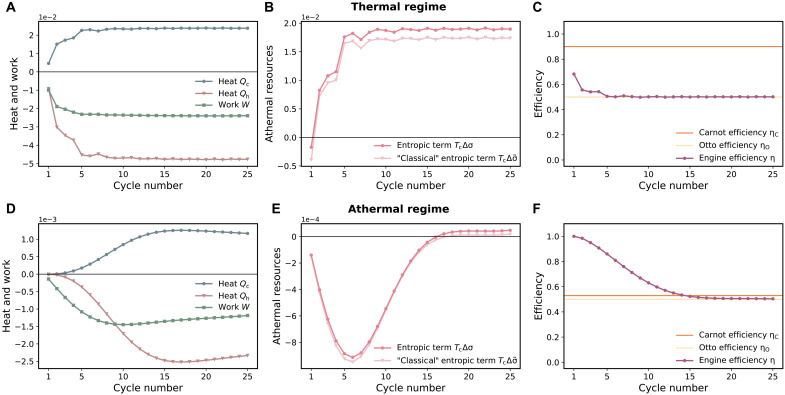
Performance of the two-oscillator engine. (**A** to **C**) In the thermal regime, the engine produces work from heat, while initial correlation are not exploited ($T_{c}\Delta \sigma >0$ after a transient). The efficiency η is always smaller than the Carnot efficiency $\eta_{C}$ and quickly converges to the Otto efficiency $\eta_{O}$. Parameters are λ=0.08, $\lambda_{c}=\lambda_{h}=\lambda \;/\;2$, $T_{c}=0.8$ and $T_{h}=8$ (corresponding to the upper right corner in Fig. 3). (**D** to **F**) In the athermal regime, the engine predominantly produces work from entropic resources such as system-bath correlations that are created during the thermalization with the baths: $T_{c}\Delta \sigma$ initially grows more negative, work is larger than the absorbed heat and the efficiency exceeds the Carnot efficiency. As the number of cycles increases, nonequilibrium entropy produces leads to $T_{c}\Delta \sigma >0$, pushing the engine to thermal operation. The “classical” entropic term $T_{c}\Delta \tilde{\sigma }$ deviates from the fully quantum expression $T_{c}\Delta \sigma$. Parameters are λ=0.08, $\lambda_{c}=\lambda_{h}=\lambda \;/\;15$, $T_{c}=0.8$, and $T_{h}=1.7$ (corresponding to the lower left corner in Fig. 3).

Figure 4 further examines the performance of the engine in the two regimes: the upper row (Fig. 4, A to C) displays the thermal case with γ=0.626 and $\eta_{\text{th}}=0.9$ (corresponding to the upper right corner of Fig. 3), whereas the lower row (Fig. 4, D to F) shows the athermal case with γ=0.02 and $\eta_{\text{th}}=0.53$ (corresponding to the lower left corner of Fig. 3). In the thermal regime, athermal resources are not used (Δσ>0 after a transient); the efficiency, which is always smaller than the Carnot efficiency, converges to the Otto efficiency $\eta_{O}=1-\omega_{c}\;/\;\omega_{h}$. By contrast, in the athermal regime, the engine initially harvests athermal resources (Δσ grows more negative during the first six cycles) and successfully converts them into work (the produced work is larger than the absorbed heat) with an efficiency that is larger than the Carnot efficiency. This effect is mainly due to the reduction of the temperature of the hot bath, which increases correlations between the latter and the second oscillator after thermalization, and at the same time decreases the heat flow between the reservoirs. However, with increasing cycle number, entropy production leads to Δσ>0, bringing the engine to thermal operation at $\eta_{O}$ after 16 cycles. In this instance, the nonequilibrium entropy σ(t+τ) produced during one cycle via quantum friction (*72*–*74*), associated with the fact that the driving Hamiltonian does not commute with the engine Hamiltonian, becomes larger than the entropic resources σ(t) generated at the beginning of each cycle; since overall more correlations are created than used in one cycle, Δσ>0 as in the thermal regime. The duration of the athermal regime could in principle be considerably extended over many more cycles by using shortcut-to-adiabaticity techniques (*75*–*82*) that suppress entropy production. Also, an alternative strategy would be to refill the “entropic tank” of the engine by letting it thermalize with the external baths to the initial state, ρ(0) = ρ_c_ ⊗ ρ_h_, before restarting it. It should be emphasized that the performance of the two-oscillator engine could not be described without the generalized laws (Eqs. 1 and 2), and the resulting efficiency (Eq. 4), highlighting the importance to extend the laws of thermodynamics to correlated machines. Last, we highlight the quantum nature of σ by replacing, for both system and baths, the relative entropy $D\left(\rho \|\rho^{\text{th}}\right)$ by the diagonal relative entropy $D\left(\rho_{d}\|\rho^{\text{th}}\right)$, where $\rho_{d}$ is the diagonal operator in the energy basis. This amounts to setting the relative entropy of coherence, $C_{r}(\rho )=S(\rho_{d})-S(\rho )$, for system and baths to zero, since $D\left(\rho \|\rho^{\text{th}}\right)=C_{r}(\rho )+D(\rho_{d}\|\rho^{\mathrm{th}})$ (*83*). The corresponding “classical” entropic term $T_{c}\Delta \tilde{\sigma }$ deviates from the fully quantum expression $T_{c}\Delta \sigma$ (Fig. 4, B and E), indicating the presence of nonclassical correlations.

## DISCUSSION

The practical usefulness of the second law is that it provides the maximum efficiency of any thermal machine. The knowledge of such upper bound is crucial for optimization purposes (*1*). We have extended the theory of engines to the correlated quantum domain by deriving exact generalized laws of thermodynamics for cyclic processes. Since no approximations are involved, we expect them to be universally applicable. The extended efficiency formula reveals that there may be efficiencies above the uncorrelated Carnot limit. This is the case in the athermal regime where engines are fueled by entropic resources, instead of heat. As we have seen, these entropic resources can occur naturally in microscopic engines. An interesting question is to determine conditions under which correlation creation outweighs entropy production, so that engines may remain in the athermal regime. In view of their generality, these findings should be relevant for the concrete design and optimization of efficient correlated quantum machines.

## MATERIALS AND METHODS

### Numerical simulations

The Hamiltonian of the two-oscillator engine simulated is

$$\begin{array}{c} \begin{array}{ll} H_{\text{tot}}\left(t\right) & \;=\frac{1}{2}\left(p_{c}^{2}+\omega_{c}^{2}x_{c}^{2}\right)+\frac{1}{2}\left(p_{h}^{2}+\omega_{h}^{2}x_{h}^{2}\right)+\lambda f\left(t\right)x_{c}x_{h}\\ & \;+\sum_{\alpha =1,\ldots ,300}\frac{1}{2}\left(\pi_{c,\alpha }^{2}+\omega_{c}^{2}q_{c,\alpha }^{2}+2\lambda_{c}q_{c,\alpha }q_{c,\alpha +1}\right)+\lambda_{c}x_{c}q_{c,1}\\ & \;+\sum_{\alpha =1,\ldots ,300}\frac{1}{2}\left(\pi_{h,\alpha }^{2}+\omega_{h}^{2}q_{h,\alpha }^{2}+2\lambda_{h}q_{h,\alpha }q_{h,\alpha +1}\right)+\lambda_{h}x_{h}q_{h,1} \end{array} \end{array}$$ (7)

where $p_{c,h}$ and $x_{c,h}$ are the momentum and position operators of the harmonic oscillators composing the working substance S, and $\pi_{c\left(h\right),\alpha }$ and $q_{c\left(h\right),\alpha }$ the ones corresponding to the oscillators of the cold (hot) reservoir ${ℛ}_{c}$ (${ℛ}_{h}$). The frequencies and the couplings were assigned the values $\omega_{c}=1$, $\omega_{h}=2$, λ=0.08, and $\lambda /15\leq \lambda_{c}=\lambda_{h}\leq \lambda /2$. Note that we used periodic boundary conditions for the reservoirs: $q_{c,301}=q_{c,1}$ and the analogous for the other operators. The bump function *f*(*t*) modulating the interaction between the two oscillators of S is given by

$$\begin{array}{c} f\left(t\right)=\left\{\begin{array}{cc} \frac{1}{2}\left\{1-\text{tanh}\left[\text{cot}\left(\mathrm{\pi t}/\delta \right)\right]\right\}\; & 0\leq t\leq \delta \\ 1\; & \delta <t<t_{\text{on}}-\delta \\ \frac{1}{2}\left\{1+\text{tanh}\left\{\text{cot}\left[\pi \left(t-t_{\text{on}}\right)/\delta \right]\right\}\right\}\; & t_{\text{on}}-\delta \leq t\leq t_{\text{on}}\\ 0\; & t_{\text{on}}<t\leq t_{\text{on}}+t_{\text{off}} \end{array}\right. \end{array}$$ (8)

and extended periodically to $t>t_{\text{on}}+t_{\text{off}}$ with period $\tau =t_{\text{on}}+t_{\text{off}}$. The on and off times used were $t_{\text{on}}=41\left[2\pi /\left(\omega_{h}-\omega_{c}\right)+1\right]/40$ and $t_{\text{off}}=5\left[2\pi /\left(\omega_{h}-\omega_{c}\right)+1\right]$. In addition, δ is the time it takes to fully switch on or off the interaction between the two osillators and was given by $\delta =0.45\;t_{\text{on}}$. The global initial state of the engine was $\rho \left(t=0\right)=\rho_{c}⊗\rho_{h}$, where

$$\begin{array}{c} \begin{array}{c} \rho_{c}=\text{exp}\left\{-\left[\frac{1}{2}\left(p_{c}^{2}+\omega_{c}^{2}x_{c}^{2}\right)+\right.\right.\\ \sum_{\alpha =1,\ldots ,300}\left.\left.\frac{1}{2}\left(\pi_{c,\alpha }^{2}+\omega_{c}^{2}q_{c,\alpha }^{2}+2\lambda_{c}q_{c,\alpha }q_{c,\alpha +1}\right)+\lambda_{c}x_{c}q_{c,1}\right]/T_{c}\right\}/Z_{c} \end{array} \end{array}$$ (9)

and

$$\begin{array}{c} \begin{array}{c} \rho_{h}=\text{exp}\left\{-\left[\frac{1}{2}\left(p_{h}^{2}+\omega_{h}^{2}x_{h}^{2}\right)+\right.\right.\\ \sum_{\alpha =1,\ldots ,300}\left.\left.\frac{1}{2}\left(\pi_{h,\alpha }^{2}+\omega_{h}^{2}q_{h,\alpha }^{2}+2\lambda_{h}q_{h,\alpha }q_{h,\alpha +1}\right)+\lambda_{h}x_{h}q_{h,1}\right]/T_{h}\right\}/Z_{h} \end{array} \end{array}$$ (10)

with $T_{c}=0.8$, $1.7\leq T_{h}\leq 8$, and $Z_{c,h}$ the corresponding normalizations.

The simulations were done using Python with a modified version of the code used in (*52*).
