# Supplementary material for: Correlated quantum machines beyond the standard second law
Source: Sci Adv. 2025 Oct 10;11(41):eadw8462. doi: 10.1126/sciadv.adw8462 (PMC13155534; doi:10.1126/sciadv.adw8462)
Supplement: Supplementary file 1 — Supplementary Text [file sciadv.adw8462_sm.pdf]

Science Advances
AAAS

Supplementary Materials for

Correlated quantum machines beyond the standard second law

Milton Aguilar and Eric Lutz

Corresponding author: Milton Aguilar, maguilar@itp1.uni-stuttgart.de

Sci. Adv. **11**, eadw8462 (2025)
DOI: 10.1126/sciadv.adw8462

This PDF file includes:

Supplementary Text

Supplementary Text

Derivation of the generalized first law of thermodynamics

In this section we derive the generalized first law of thermodynamics (Eq. 1 of the main text):

$$W = \sum_j Q_j + \Delta U, \tag{S1}$$

where $W = \int_t^{t+\tau} dt' \langle \partial_{t'} H_{\mathrm{tot}}(t') \rangle$ is the total work extracted during a cycle of duration τ, $Q_j = \langle H_j \rangle (t+\tau) - \langle H_j \rangle (t)$ is the heat absorbed by reservoir $\mathcal{R}_j$, and $\Delta U = \sum_i \Delta U_i = \sum_i [\langle H_i + \sum_j H_{ij} \rangle (t+\tau) - \langle H_i + \sum_j H_{ij} \rangle (t)]$ is the change of the energy of the compound system, including the interaction energy. To this end, we remind the reader that the total Hamiltonian is given by

$$H_{\mathrm{tot}}(t) = \sum_i H_i(t) + \sum_j H_j + \sum_{i,j} H_{ij}(t), \tag{S2}$$

with $H_i(t)$ the Hamiltonian of the subsystem $\mathcal{S}_i$, H_j the Hamiltonian of the reservoir $\mathcal{R}_j$, and $H_{ij}(t)$ the interaction Hamiltonian between $\mathcal{S}_i$ and $\mathcal{R}_j$. On the one hand, the generalized version of Ehrenfest theorem implies

$$\frac{d}{dt} \langle H_{\mathrm{tot}} \rangle = \langle \partial_t H_{\mathrm{tot}}(t) \rangle. \tag{S3}$$

On the other hand, due to the linearity of differentiation, we have

$$\frac{d}{dt} \langle H_{\mathrm{tot}} \rangle = \sum_i \frac{d}{dt} \langle H_i \rangle + \sum_{i,j} \frac{d}{dt} \langle H_{ij} \rangle + \sum_j \frac{d}{dt} \langle H_j \rangle. \tag{S4}$$

Combining Eqs. (S3) and (S4), we further obtain

$$\langle \partial_t H_{\mathrm{tot}}(t) \rangle = \sum_i \frac{d}{dt} \langle H_i + \sum_j H_{ij} \rangle + \sum_j \frac{d}{dt} \langle H_j \rangle. \tag{S5}$$

By integrating with respect to t, we finally arrive at the generalized first law of thermodynamics:

$$\begin{aligned} \underbrace{\int_t^{t+\tau} dt' \langle \partial_{t'} H_{\mathrm{tot}}(t') \rangle}_{W} &= \underbrace{\sum_i [\langle H_i + \sum_j H_{ij} \rangle (t+\tau) - \langle H_i + \sum_j H_{ij} \rangle (t)]}_{\Delta U} \\ &\quad + \underbrace{\sum_j [\langle H_j \rangle (t+\tau) - \langle H_j \rangle (t)]}_{Q_j}. \end{aligned} \tag{S6}$$

Note that

$$\langle \partial_t H_{\text{tot}}(t) \rangle = \sum_i \langle \partial_t H_i(t) \rangle + \sum_{i,j} \langle \partial_t H_{ij}(t) \rangle \tag{S7}$$

and

$$\frac{d}{dt}\langle H_{ij} \rangle = \frac{1}{i\hbar}\langle [H_{ij}(t), H_i(t) + H_j] \rangle + \langle \partial_t H_{ij}(t) \rangle. \tag{S8}$$

Thus, Eq. (S6) can be rewritten as

$$\begin{aligned} \underbrace{\sum_i \int_t^{t+\tau} dt' \langle \partial_{t'} H_i(t') \rangle}_{W_S} = \underbrace{\sum_j [\langle H_j \rangle (t+\tau) - \langle H_j \rangle (t)]}_{Q_j} + \underbrace{\sum_i [\langle H_i \rangle (t+\tau) - \langle H_i \rangle (t)]}_{\Delta U_S} \\ + \sum_{i,j} \int_t^{t+\tau} dt' \langle [H_{ij}(t'), H_i(t') + H_j] \rangle. \end{aligned} \tag{S9}$$

Therefore, if $\Delta U_{\mathcal{S}} = 0$ and the interactions are energy-conserving on average, $\sum_{i,j} \int_t^{t+\tau} dt' \langle [H_{ij}(t'), H_i(t') + H_j] \rangle = 0$, the standard version of the first law of thermodynamics is then recovered: $W_{\mathcal{S}} = \sum_j Q_j$.

Derivation of the generalized second law of thermodynamics

In this section we derive the generalized second law of thermodynamics (Eq. 2 of the main text):

$$\sum_i \Delta S(\rho_i) + \sum_j \frac{Q_j}{kT_j} = \Delta\Sigma, \tag{S10}$$

where k is the Boltzmann constant, $\rho_i = \mathrm{tr}_{\bar{\mathcal{S}}_i}(\rho_{\mathcal{S}\mathcal{R}})$ is the reduced state of the subsystem $\mathcal{S}_i$ ($\bar{\mathcal{S}}_i$ is the complement of $\mathcal{S}_i$), $\Delta S(\rho_i) = S(\rho_i(t+\tau)) - S(\rho_i(t))$ with $S(\rho) = -\mathrm{tr}[\rho \ln(\rho)]$ the von Neumann entropy, and $\Delta\Sigma = \Sigma(t+\tau) - \Sigma(t)$ with $\Sigma = I(\mathcal{S},\mathcal{R}) + C(\mathcal{S}) + C(\mathcal{R}) + \sum_j D(\rho_j || \rho_j^{\text{th}})$. Additionally, $I(\mathcal{S},\mathcal{R}) = S(\rho_{\mathcal{S}}) + S(\rho_{\mathcal{R}}) - S(\rho_{\mathcal{S}\mathcal{R}})$ is the mutual information between the system $\mathcal{S}$ and the collection of all reservoirs $\mathcal{R}$, and $C(\mathcal{S}) = \sum_i S(\rho_i) - S(\rho_{\mathcal{S}})$ and $C(\mathcal{R}) = \sum_j S(\rho_j) - S(\rho_{\mathcal{R}})$ are the total correlations between all the subsystems and all the reservoirs, respectively. Furthermore, $D(\rho_j || \rho_j^{\text{th}})$ is the relative entropy between the reduced state of $\mathcal{R}_j$, ρ_j, and the reference thermal

state $\rho_j^{\text{th}} = \exp(-H_j/kT_j)/Z_j$. We begin by showing the validity of the following identity:

$$\begin{aligned}
\sum_i \Delta S(\rho_i) + \sum_j \Delta S(\rho_j) &= \Delta I(\mathcal{S},\mathcal{R}) + \Delta C(\mathcal{S}) + \Delta C(\mathcal{R}) \\
&= \underbrace{[\Delta S(\rho_{\mathcal{S}}) + \Delta S(\rho_{\mathcal{R}}) - \Delta S(\rho_{\mathcal{SR}})]}_{\Delta I(\mathcal{S},\mathcal{R})} + \underbrace{\left[\sum_i \Delta S(\rho_i) - \Delta S(\rho_{\mathcal{S}})\right]}_{\Delta C(\mathcal{S})} \\
&+ \underbrace{\left[\sum_j \Delta S(\rho_j) - \Delta S(\rho_{\mathcal{R}})\right]}_{\Delta C(\mathcal{R})} \\
&= \sum_i \Delta S(\rho_i) + \sum_j \Delta S(\rho_j) + \underbrace{[\Delta S(\rho_{\mathcal{S}}) - \Delta S(\rho_{\mathcal{S}})]}_{=0} \\
&+ \underbrace{[\Delta S(\rho_{\mathcal{R}}) - \Delta S(\rho_{\mathcal{R}})]}_{=0} - \underbrace{\Delta S(\rho_{\mathcal{SR}})}_{=0} \\
&= \sum_i \Delta S(\rho_i) + \sum_j \Delta S(\rho_j).
\end{aligned} \tag{S11}$$

We next write the relative entropy $D(\rho_j||\rho_j^{\text{th}})$ explicitly as

$$D(\rho_j(t)||\rho_j^{\text{th}}) = \text{tr}[\rho_j \ln(\rho_j)] - \text{tr}[\rho_j \ln(\rho_j^{\text{th}})] = -S(\rho_j) + \frac{1}{kT_j}\text{tr}[\rho_j(t)H_j] + \ln(Z_j), \tag{S12}$$

which implies

$$\Delta S(\rho_j) = \frac{Q_j}{kT_j} - \Delta D(\rho_j||\rho_j^{\text{th}}). \tag{S13}$$

Finally, we replace Eq. (S13) in the identity in Eq. (S11) to obtain the generalized second law of thermodynamics:

$$\sum_i \Delta S(\rho_i) + \sum_j \frac{Q_j}{kT_j} = \underbrace{\Delta I(\mathcal{S},\mathcal{R}) + \Delta C(\mathcal{S}) + \Delta C(\mathcal{R}) + \sum_j \Delta D(\rho_j||\rho_j^{\text{th}})}_{\Delta\Sigma}. \tag{S14}$$

Derivation of the generalized efficiency

Let us next derive a generalized formula for the efficiency of any quantum engine (Eq. 4 of the main text):

$$\eta = \gamma\left(\eta_{\text{th}} - \frac{kT_{\text{min}}\Delta\sigma}{Q^{\text{in}}}\right), \tag{S15}$$

where $\gamma = [1 + \Delta U^{\text{in}}/Q^{\text{in}}]^{-1}$ and $\eta_{\text{th}} = -\sum_j \eta_j Q_j/Q^{\text{in}}$, with $Q^{\text{in}} = \sum_j (|Q_j| - Q_j)/2$ and $\Delta U^{\text{in}} = \sum_i (|\Delta U_i| - \Delta U_i)/2$. To this end, we first rewrite the first law of thermodynamics as

$$
\begin{aligned}
W &= \sum_j Q_j + \sum_i kT_{\text{min}}\Delta S(\rho_i) + \sum_i [\Delta U_i - kT_{\text{min}}\Delta S(\rho_i)] \\
&= \sum_j Q_j + \sum_i kT_{\text{min}}\Delta S(\rho_i) + \sum_i \Delta F_i
\end{aligned}
\tag{S16}
$$

with $F_i = U_i - kT_{\text{min}} S(\rho_i)$ the generalized free energy. On the other hand, from the second law of thermodynamics we know

$$
\sum_i \Delta S(\rho_i) = -\sum_j \frac{Q_j}{kT_j} + \Delta\Sigma. \tag{S17}
$$

We now combine the two previous equations to obtain Eq. 3 of the main text:

$$
W = \sum_j \underbrace{\left(1 - \frac{T_{\text{min}}}{T_j}\right)}_{\eta_j} Q_j + \underbrace{kT_{\text{min}}\Delta\Sigma + \sum_i \Delta F_i}_{kT_{\text{min}}\Delta\sigma} \tag{S18}
$$

Finally, we compute the efficiency η with the help of Eq. (S18) and find

$$
\eta = -\frac{W}{Q^{\text{in}} + \Delta U^{\text{in}}} = -\underbrace{\frac{1}{1 + \frac{\Delta U^{\text{in}}}{Q^{\text{in}}}}}_{\gamma} \frac{W}{Q^{\text{in}}} = \gamma \left(\underbrace{-\sum_j \eta_j \frac{Q_j}{Q^{\text{in}}}}_{\eta_{\text{th}}} - \frac{kT_{\text{min}}\Delta\sigma}{Q^{\text{in}}} \right). \tag{S19}
$$

Comparison with a previous approach

It is instructive to compare our exact findings with those obtained using different approaches. We shall concretely compare our generalized second law with the generalized Clausius inequality for correlated systems obtained using an information-theoretic approach in Ref. (13) (see Eq. (8) in that article). This result is based on a number of assumptions, in particular, an entropy preserving oper-ation between two infinite-sized thermal baths A and B which leaves their respective Hamiltonians unchanged. Assuming further that such operation is energy non-increasing and that the initial state of the baths is an uncorrelated product of thermal states, the heat flow between the baths is found to obey the generalized Clausius inequality

$$
-\tilde{Q}_A(T_B - T_A) \geq kT_A T_B \Delta I(\mathcal{R}_A, \mathcal{R}_b), \tag{S20}
$$

where $T_{A,B}$ are the initial bath temperatures, $I(\mathcal{R}_A, \mathcal{R}_b)$ is the mutual information between them, and $\tilde{Q}_A = -kT_A \Delta S(\rho_A)$ is the heat (with S the von Neumann entropy).

Equation (S20) can be recovered, and extended, starting from our exact first and second laws, Eqs. (1) and (2) of the main text. We model direct contact between the two reservoirs by considering a working substance whose state is cyclic, whose Hamiltonian is constant in time, and that does not get correlated with the two baths. In that way, the working substance does not disturb the states of the baths, and only acts as a connection between them. Setting moreover $\Delta U_{\text{int}} = 0$, we find from our generalized first and second laws

$$Q_A(T_B - T_A) = kT_A T_B \left[\Delta I(\mathcal{R}_A, \mathcal{R}_b) + D(\rho_A || \rho_A^{\text{th}}) + D(\rho_B || \rho_B^{\text{th}})\right]. \tag{S21}$$

We first observe that, since the relative entropies are positive, we have $Q_A\,(T_B - T_A) \geq kT_A T_B \Delta I\,(\mathcal{R}_A, \mathcal{R}_A)$. We hence recover an inequality for the heat that is similar to that of Ref. (13). However, the two definitions of heat differ: ours is based on energy changes, while that of Ref. (13) is based on entropy changes. Both definitions can be related by noting that, since the initial state of the reservoirs is thermal, $D(\rho_A || \rho_A^{\text{th}}) = \Delta D(\rho_A || \rho_A^{\text{th}}) = Q_A/kT_A - \Delta S(\rho_A) = (Q_A + \tilde{Q}_A)/kT_A$. In other words, $Q_A = -\tilde{Q}_A + kT_A \Delta D(\rho_A || \rho_A^{\text{th}})$, showing that both expressions agree at equilibrium (except for the sign). Using the above equality and adding $T_A \tilde{Q}_A$ to both sides, we obtain the exact equality

$$-\tilde{Q}_A(T_B - T_A) = kT_A T_B \Delta I(\mathcal{R}_A, \mathcal{R}_b) + kT_A \left[T_A \Delta D(\rho_A || \rho_A^{\text{th}}) + T_B \Delta D(\rho_B || \rho_B^{\text{th}})\right], \tag{S22}$$

which generalizes the inequality (S20) of Ref. (13).
